# Concise Review: The Regenerative Journey of Pericytes Toward Clinical Translation

**DOI:** 10.1002/stem.2846

**Published:** 2018-05-31

**Authors:** William Cathery, Ashton Faulkner, Davide Maselli, Paolo Madeddu

**Affiliations:** ^1^ Experimental Cardiovascular Medicine, University of Bristol, Bristol Heart Institute, Bristol Royal Infirmary Bristol United Kingdom; ^2^ School of Bioscience and Medicine, University of Surrey, Guildford, United Kingdom & IRCCS Multimedica Milan Italy

**Keywords:** Pericytes, Translational medicine, Cell therapy, Coronary artery disease, Regenerative medicine

## Abstract

Coronary artery disease (CAD) is the single leading cause of death worldwide. Advances in treatment and management have significantly improved patient outcomes. On the other hand, although mortality rates have decreased, more people are left with sequelae that require additional treatment and hospitalization. Moreover, patients with severe nonrevascularizable CAD remain with only the option of heart transplantation, which is limited by the shortage of suitable donors. In recent years, cell‐based regenerative therapy has emerged as a possible alternative treatment, with several regenerative medicinal products already in the clinical phase of development and others emerging as competitive preclinical solutions. Recent evidence indicates that pericytes, the mural cells of blood microvessels, represent a promising therapeutic candidate. Pericytes are abundant in the human body, play an active role in angiogenesis, vessel stabilization and blood flow regulation, and possess the capacity to differentiate into multiple cells of the mesenchymal lineage. Moreover, early studies suggest a robustness to hypoxic insult, making them uniquely equipped to withstand the ischemic microenvironment. This review summarizes the rationale behind pericyte‐based cell therapy and the progress that has been made toward its clinical application. We present the different sources of pericytes and the case for harvesting them from tissue leftovers of cardiovascular surgery. We also discuss the healing potential of pericytes in preclinical animal models of myocardial ischemia (MI) and current practices to upgrade the production protocol for translation to the clinic. Standardization of these procedures is of utmost importance, as lack of uniformity in cell manufacturing may influence clinical outcome. Stem Cells *2018;36:1295–1310*

**Significance Statement**

Pericytes show great promise for the treatment of coronary artery disease; however, despite recent progress, research that has been translated to the clinic is lacking. This review summarizes the journey of pericytes from bench to bedside, evaluating the progress and potential that has been demonstrated so far, and the considerations that will need to be taken on board before clinical translation becomes a reality.

## Introduction

Coronary artery disease (CAD) is the leading cause of death worldwide and in the U.K. alone is responsible for approximately 70,000 deaths each year 1. Of those that survive, many go on to develop heart failure (HF) as myocardial performance continues to decline. A particular problem is posed by those patients presenting with ST‐elevation myocardiaI infarction (STEMI) who are not amenable to revascularization or receive revascularization later than recommended. This results in larger infarcts and an increased risk of HF. There is no viable treatment for post‐ischemic end‐stage HF patients, apart from heart transplantation. However, these are of limited supply and pose additional complications 2. In addition, there is growing number of patients who manifest angina attacks that cannot be controlled by optimal medical treatment or revascularization. These patients may have a preserved cardiac contractility but suffer a severe limitation in physical activities, which compromises their quality of life and productivity, thereby translating into increased social costs. In the United States, there are ≈850,000 people who suffer refractory angina, with this figure being mirrored in Europe by the occurrence of 100,000 new cases per year 3. It is now well recognized that these patients have coronary microvascular disease, with impaired endothelium‐mediated vasorelaxation and reduced blood flow reserve. New and improved treatments that go beyond reducing cardiovascular risk factors and toward true cardiovascular repair are clearly needed.

In recent years, advancement in our understanding of stem cells and their regenerative capacity has presented an alternative treatment strategy with the potential for recovering lost heart function. However, the clinical application of such treatment has so far yielded a success inferior to the initial promises 4, 5, 6, 7. The majority of trials to date have involved the delivery of bone marrow‐derived cell populations; however, the use of alternative cardiovascular‐derived cell sources that perhaps hold greater applicability for myocardial repair are now coming to the forefront. Pericytes represent a new entry in the growing list of medicinal cell products. These cells, found within the perivascular region of blood vessels in close contact with the endothelium, are principally thought to take up a supportive role to the aligning endothelium, acting to stabilize the vessel, regulate microvascular blood flow and facilitate angiogenesis 8.

This review will discuss the potential of autologous pericytes as a model of a bench‐to‐bedside cell therapy approach for the treatment for CAD. Particular emphasis will be placed on the identification of pericytes regenerative potential, the protocols for pericyte isolation, expansion and prospective delivery to patients, and the progress that has been made toward clinical translation.

## What Constitutes a Pericyte?

Pericytes were initially defined by their anatomical location, encircling the endothelium of microvascular capillaries, terminal arterioles, and post‐capillary venules 9. They can be found within most tissues of the body; however, their morphology, biology, and density vary between organs depending on the stringency of the endothelial barrier properties. For example, the pericyte to endothelial cell ratio can be as great as 1:100 within the skeletal muscle through to 1:3 and 1:1 in the central nervous system and retina, respectively, regions where vessel integrity and trans‐endothelial movement is tightly regulated 10.

Phenotypically, pericytes express a range of antigenic markers that help with their identification. No single antigen can be regarded as being pericyte‐specific, meaning their identification is based on a combination of markers. Commonly, these include neural/glial antigen 2 (NG2) proteoglycan, platelet‐derived growth factor receptor‐β (PDGFRβ), and CD146 (Table 1), together with mesenchymal markers, such as CD90 and CD105, but absence of CD56, a surface antigen expressed in neurons, glia and skeletal muscle, and hematopoietic and endothelial markers, such as CD45 and CD31, respectively. It is worth keeping in mind, however, that due to the heterogeneity of these cells and lack of an agreed phenotype, different research groups have reported the presence of pericytes within the same tissue but with conflicting antigenic profiles 11, 12.

**Table 1:** Antigenic markers commonly used to identify pericytes from different organs and anatomical locations

Marker	Function	Expression	Possible explanation for expression	References
**NG2 (Neural/glial antigen 2)**	Membrane proteoglycan that mediates cell‐cell and cell‐extracellular matrix interactions.	Positive in pericytes of arterioles and capillaries as well as vasa vasorum, however, negative in venule pericytes.	NG2 contributes to transmembrane signaling and has been linked to promotion of cell proliferation and motility. It is, therefore, not surprising that this is expressed in pericytes, a highly mobile and proliferative cell type. NG2 has also been suggested to play a role in vascular network homeostasis, with its absence in venous vessels contributing to regulation of arterial/venous anastomoses.	13, 14, 15, 16, 17
**PDGFRβ (Platelet‐derived growth factor receptor‐beta)**	Tyrosine‐protein kinase receptor that mediates the differentiation of pericyte progenitor cells.	Ubiquitous marker of micro vessel and adventitial pericytes	During angiogenesis, vessel stabilization is achieved via pericyte recruitment. This is achieved via PDGF‐β signaling and, therefore, it is essential for pericytes to express the receptor for this, PDGFR‐β.	15, 18, 19, 20
**CD146 (Melanoma cell adhesion molecule or MCAM)**	Membrane glycoprotein involved in heterophilic cell‐cell interactions.	Marker of brain, bone marrow, myocardial and skeletal muscle pericytes. Negative in adventitial pericytes.	CD146 has been shown to regulate PDGFRβ pericyte endothelial signaling in the blood‐brain barrier development. CD146 has also been suggested as a marker for multipotency which explains its presence in pericytes. The absence of this marker in adventitial pericytes has not been explored.	12, 13, 15, 21, 22, 23, 24, 25
**CD13 (Aminopeptidase N)**	Aminopeptidase N is a membrane type II metalloprotease. It is implicated in cell migration, cell survival and angiogenesis.	Marker of cerebral pericytes associated with the blood—brain barrier	It is thought that pericytic aminopeptidase N is involved in metabolism of neurotransmitter in the blood brain barrier and is therefore restricted to cerebral pericytes.	18, 26, 27
**αSMA (Alpha‐smooth muscle actin)**	Highly conserved contractile protein involved in cell motility, structure, integrity, and intercellular signaling.	Pericytes express αSMA at a concentration of one tenth of smooth muscle cells expression, but sixfold higher than endothelial cells. They can increase their expression in response to stress or vascular remodeling.	αSMA is a crucial contractile protein involved in vasoconstriction. Pericytes control blood flow in capillaries via an active response which requires expression of contractile proteins. This expression is most likely lower than smooth muscle cells as pressure in capillaries is lower than the arterial system which require greater contraction.	15, 28, 29, 30
**Nestin**	Intermediate filament of the cytoskeleton involved in the remodeling of the cell.	Markers of a subpopulation of pericytes in brain, bone marrow, liver, and skeletal muscle that shows multipotential regenerative ability.	Nestin was originally described as a neural progenitor marker; however, studies have suggested a link between nestin expression and neovascularization providing a possible explantation for pericytic expression.	13, 31, 32, 33, 34
**ALP (Alkaline‐phosphatase)**	Enzyme found in the blood that plays an integral role in metabolism in the liver.	In vivo marker expressed across different pericyte subsets, with notable expression in skeletal pericytes.	The physiological function of ALP remains obscure with little description in the literature. Pericyte expression and locality to blood vessels could indicate a role in the release of ALP into the bloodstream.	23, 35, 36
**CD34**	Transmembrane phosphoglycoprotein thought to play a role in cytoadhesion, and regulation of differentiation and proliferation	In the absence of CD31, a marker for endothelial cells, which also express CD34, expression of this antigen acts as a marker for a subpopulation of adventitial pericytes.	The function of CD34 as a surface antigen is still unknown; however, it is linked to stem cell and progenitor activity, and pronounced differentiation capacity, which may account for expression in certain pericytes.	12, 22, 37

More recently, cells with pericyte‐like properties have also been described within the wall of larger vessels, thereby challenging the original concept of pericytes localizing only to the microvasculature. For example, we were the first to describe such cells present around the adventitial vasa vasorum of the human saphenous vein 22. These cells are CD34 positive but CD31, CD146 and CD45 negative. At the same time, they also co‐express typical pericyte markers including NG2, PDGFRβ, CD105 and CD90. Corselli et al. have also demonstrated the presence of CD34^+^/CD146^–^/CD31^–^ cells around larger blood vessels of multiple organs that co‐express pericyte markers in culture following exposure to vascular growth factors 38.

Due to the absence of a unique marker, tracking pericyte lineage has traditionally proven difficult. So far, studies have suggested pericytes develop from either the ectoderm or mesoderm, depending on their anatomical location. More specifically, through use of neural crest fate mapping models, it has been shown that pericytes in the CNS, thymus, retina, and choroid have developed from differentiated neural crest‐derived cells 39, 40. On the other hand, as summarized by Armulik et al, pericytes found in coelomic organs, such as the lungs, liver, or coronary vessels, have been identified as mesothelium derived 18. Here it has been suggested that mesothelial cells undergo an epithelial‐to‐mesenchymal transition, followed by a migration to a specific organ and differentiation into pericytes 18. To further add to the complexity of these cells, recent publications have suggested that pericytes from the same tissue can have a heterogenous origin 41, 42. For example, Chen et al demonstrated coronary pericytes also arise from endocardial cells undergoing endothelial‐to‐mesenchymal transition, and some retinal pericytes have been shown to be bone‐marrow derived in addition to some originating in the neural crest 43. It is clear there is still a lack of understanding regarding pericyte ontogeny and development. More work to identify the origin of a type of pericyte could help identify their regenerative potential for particular pathologies. For example, ensuring pericytes used for treatment are derived from the same germ layer as the transplant site could improve beneficial effects.

## Exploring the Regenerative Potential of Pericytes

Pericytes are a multifunctional cell type that play a commanding role in maintaining homeostasis. Previous studies have identified their involvement in many physiologic processes, including modulation of immune response, vascular development, regulation of blood flow, stabilization of vessels, and contribution to the endothelial barrier integrity 9, 44, 45. Below we summarize the role of pericytes in some of these processes that are of particular importance to regenerative medicine.

### Angiogenesis

The stimulation of angiogenesis, for example, the formation of new vessels from the pre‐existing vasculature, is an essential yet challenging requirement for tissue repair 46, 47. It is important, therefore, that the chosen stem cell population targeted for use in the treatment of ischemic disease can actively engage with the angiogenic process in order to stimulate the outgrowth of mature and functional neo‐vessels. Pericytes address this requirement by playing an active role during both the vessel sprouting and stabilization phases of angiogenesis.

Neovascular formation is initiated by the activation of quiescent vessels in response to angiogenic signals, such as vascular endothelial growth factor (VEGF), angiopoietin 2 (ANG‐2), or chemokines. ANG‐2, which is almost exclusively expressed by endothelial cells (ECs), promotes detachment and migration of pericytes from the endothelial layer. This action is carried out via the inhibition of Tie2 receptors. Historically, ANG‐2 was thought to be an autocrine modulator of the ANG/Tie signaling pathway. However, recent studies have demonstrated Tie2 receptor expression on pericytes as well as ECs 48. The identification of these Tie2 receptors highlights the importance of pericytes for angiogenesis and vessel stabilization. Pericyte expression of ANG‐1, in the absence of ANG‐2, activates both pericyte and EC Tie2 receptors, triggering downstream pathways that contribute to vascular maturation. However, in the presence of ANG‐2, the Tie2 receptors are inhibited, promoting cell migration and new angiogenic activities. This is also evidenced in the recent study by Augustin and coworkers, who demonstrated pronounced activation of angiogenesis following the silencing of pericyte Tie2 48.

To aid detachment of pericytes and EC migration, both cell types secrete matrix metalloproteases (MMPs) which degrade the basement membrane 49. After detachment, pericytes change their quiescent phenotype, shorten their processes, increase in volume and begin to proliferate 8. Meanwhile, ECs loosen their junctions, which, in combination with the action of VEGF, increases the permeability of the endothelial layer and allows passage of plasma proteins which lay down extracellular matrix (ECM) 49. ECs migrate outward into the new ECM in response to angiogenic factors. They are led by a single EC with high migration and low proliferation rates, known as a “tip cell,” which migrates toward a VEGF gradient 50. This VEGF signaling is spatially restricted via pericyte expression of VEGF receptor 1 (VEGFR1) 51. Neighboring ECs, called “stalk cells,” fall in behind the tip cell and form the lumen as the growing sprout extends into the avascular area 49. Eventually the sprouting branch fuses with a neighboring branch to form a primitive vessel. In order to stabilize these primitive vessels, pericytes are recruited via signals such as PDGF‐β and PDGF‐B 19, 20. Both the newly recruited pericytes and ECs facilitate the maturation process via secretion of paracrine factors, such as transforming growth factor‐beta (TGFβ) and ANG‐1, which promote pericyte re‐attachment and endothelial barrier formation, while at the same time suppressing EC proliferation and migration 18, 52.

### Differentiation

Pericytes are a multipotent cell type that share part of their origin with mesenchymal stromal cells (MSCs) 53. They can trans‐differentiate into typical cells of the mesenchymal lineage, such as adipocytes, chondrocytes, osteocytes, myocytes, and neural cells 54. It is thought that this ability to differentiate into multiple cell types may contribute to regenerative mechanisms following tissue injury or disease 44. For example, several studies show the ability of pericytes to differentiate into immune cells, such as dendritic cells and macrophage‐like cells, which play an important role in mediating inflammation under pathological conditions 55, 56. It is also known that pericytes demonstrate a strong activated response to ischemia/hypoxia. Following an ischemic stroke, cerebral pericytes may differentiate into neural cells, vascular cells, and microglia, producing all the components of the neurovascular unit (NVU) 57.

Pericytes from different muscular tissues also show a wide differentiation potential in vitro and in vivo, while still retaining some specificity relating to their tissue of origin. For example, pericytes resident in skeletal muscle can contribute to myofiber regeneration 58. Dellavalle et al. showed that human skeletal muscle pericytes transplanted into dystrophic immunodeficient mice could generate myofibers expressing human mini‐dystrophin 59. In addition, microvascular pericytes within the human myocardium exhibit angiogenic behavior in response to hypoxia and seemly have a discernible, though modest, cardiomyogenic potential in vivo 11. However, data from our group indicate a more restricted fate of pericytes from the human heart and vasculature, with specific commitment to the vascular smooth muscle cell (VSMC) lineage 12, 22.

More recently, a study using gene tracking in developing mice has documented the potential of epicardial pericytes being the source of coronary mural cells 60. This opens new and exciting horizons for either pharmacologically activating resident pericytes, or transplanting autologous pericytes within the infarcted heart, in order to induce reparative activity, including that of arteriogenesis. It should be noted, however, that a recent study using lineage tracing of cells expressing the transcription factor *Tbx18*, which labels most pericytes more specifically than PDGFRβ, has questioned the current view of endogenous pericytes as tissue‐resident progenitors. It is instead suggested that the plasticity observed in vitro or following transplantation in vivo arises from artificial cell manipulations ex vivo 61. It cannot be excluded that the progenitor cell property is restricted to a limited number of early pericytes which do not express Tbx18.

### Regulation of Blood Flow

Similar to VSMCs, pericytes express contractile‐related proteins such as alpha smooth muscle actin (α‐SMA), myosin, tropomyosin, and vimentin 62. It is thought that because of these contractile proteins, together with their location around capillaries, pericytes are able to control blood flow in the microvasculature. In the retina, cerebellum, and cerebral cortex, pericytes have been shown to modify capillary diameter in reaction to depolarization, neurotransmitter action or neuronal activity 63. This was shown to be an active response as capillary contraction was observed before arteriole contraction 64. Contrary to this, however, Hill et al. described VSMCs as being responsible for the control of blood flow 65. In a recent review 63, it was acknowledged that the same cells were described in both studies but with different methods to classify cells as either pericytes or VSMCs. Regardless of the issue of classification, it must be noted that evidence for pericyte regulation of blood flow is mainly limited to pericytes in the brain. To fully determine their regulatory function, pericytes from other anatomical sites should be studied.

### Immunomodulation

The ability of pericytes to regulate the immune response is an important property for a regenerative cell type as it opens up the possibility of allogenic cell therapy. The major reason for transplant rejection is the response of the adaptive immune system to alloantigens through activation of T cells 66. Activation of T cells is regulated via three signals: antigen presentation via cell surface major histocompatibility complex (MHC) class I or II stimulatory molecules, costimulation with costimulatory molecules, and secretion of cytokines 66. With regard to T cell activation pathways, pericytes from various sources have been reported to be poorly immunogenic 66, 67. The pericytes did not basely express MHC class II molecules, such as HLA‐DR, nor costimulatory molecules CD80/CD86 67. Instead pericytes were found to mediate the formation of suppressive allogeneic CD4 + CD25highFoxP3 + CD1272 Tregs, regulatory T cells that maintain immunologic self‐tolerance, in a TGF‐b‐dependent and PD‐L1‐dependent manner 66. In addition, even after induction of class I and II MHC molecules, pericytes were unable to stimulate allogeneic CD4 T cell proliferation or cytokine release, and in fact rendered the T cells unresponsive to endothelial cells of the same donor 67. This behavior indicates the possibility of using pericytes in allogenic stem cell therapy.

T cell inhibition has also been identified in retinal pericytes 68. Here it was demonstrated that retinal pericytes were able to significantly inhibit active T cell proliferation and inflammatory cytokine production. This inhibition was activated through both cell‐cell contact and release of factors such as PD‐L1 and IL‐10. In addition to this, the retinal pericytes were able to reduce inflammation induced apoptosis in neighboring endothelial cells 68.

## Pericyte Metabolism and the Ischemic Environment

To obtain optimal functional activity from transplanted pericytes, their behavior under ischemia needs to be better understood. At the center of the ischemic microenvironment is a severe disturbance in metabolic homeostasis, characterized by a limited availability in nutrients and oxygen (hypoxia). Any cell transplanted into this region must, therefore, demonstrate a level of metabolic flexibility that allows the cell to survive and remain capable of eliciting a functional response. Early indications suggest that pericytes may offer a sufficient degree of metabolic resistance that may add to their therapeutic potential 22, 69, 70, 71, 72, 73.

Phenotypic characterization of pericyte metabolism is limited, and to date, has principally focused on those of the retinal and neurovascular regions 70, 74, 75. Similar to ECs, pericytes predominantly express the non‐insulin dependent glucose transporter, GLUT1, and show preference for utilizing glycolysis to support their basal metabolic needs rather than mitochondrial oxidative phosphorylation (OXPHOS) 74, 75, 76. This is in line with an oxygen consumption rate being far lower than that measured for many other cell types 77. Additionally, pericytes show a relatively modest response to the ATP synthase inhibitor, oligomycin, suggesting limited reliance on mitochondrial ATP production, although the relatively high mitochondrial reserve capacity observed in these cells suggests that they are only working at a fraction of their capacity 75. Whether this is true for all pericytes or, moreover, whether this is altered following their activation has not been determined.

An advantage of having a low oxidative rate is that it would allow pericytes to carry out their pro‐angiogenic function within tissue regions, including low perfused areas of the heart, where oxygen is limiting. In parallel, this would preserve oxygen availability to underlying and more energy‐demanding cells, such as cardiomyocytes, and limit local OXPHOS‐induced ROS production. Such a metabolic phenotype may also explain why pericytes from a range of tissue sources appear to be conferred with the ability to withstand hypoxic insult in vitro, and survive transplantation in rodent models of myocardial and peripheral ischemia 22, 69, 70, 71, 72, 78. Moreover, we have shown that adventitial pericytes (APCs) isolated from the saphenous vein have substantial levels of the anti‐oxidant enzymes superoxide dismutase (SOD) and catalase, whilst at the same time containing less ROS‐generating NADPH oxidase 4 (NOX4), particularly when compared with ECs 73. When this is disrupted, through silencing of *SOD3*, APCs lose their ability to restore blood flow upon transplantation into the ischemic hindlimb 73. This not only suggests that APCs have an enhanced resistance to oxidative stress, but moreover, that the ability to preserve a low level of intracellular ROS is essential to maintaining their functional, and thereby therapeutic, ability. Whether such characteristics are conserved across pericytes from various tissues has yet to be fully investigated.

Not only can pericytes withstand hypoxia but they also appear to positively respond to such an environment by upregulating the expression and secretion of angiogenic factors 70, 71, 72, 79. For example, we have shown that APCs release VEGF‐A, ANG‐1, and the microRNA‐132 (miR‐132) upon exposure to hypoxia, all of which act on ECs to facilitate pro‐angiogenic behavior 72. Indeed, preventing the upregulation and secretion of miR‐132, by pre‐treating APCs with anti‐miR‐132 silencing sequences prior to transplantation, significantly reduces the capacity of APCs to improve cardiac contractility, reparative angiogenesis and interstitial fibrosis in the infarcted rodent heart 72. A similar hypoxic response has also been described in microvascular pericytes (CD146^+^/CD34^–^/CD45^–^/CD56^–^) isolated from human skeletal muscle 71. In response to hypoxia in vitro, these cells significantly increase the expression and secretion of angiogenic factors (VEGF‐A, PDGF‐β, and TGF‐β1) and exert an anti‐proliferative action on cardiac fibroblasts. Furthermore, the transplantation of these skeletal muscle microvascular pericytes into the infarcted mouse heart was associated with an angiogenic and anti‐fibrotic response, leading to improvements in cardiac function 71. In both instances, the precise mechanisms by which hypoxia induced such positive effects have yet to be identified but is likely to involve oxygen‐sensitive pathways, such as the hypoxia‐inducible transcription factor, HIF1α, as indicated by others 69, 80.

Overall, despite a limited number of studies conducted to date, pericytes appear reliant on a metabolic co‐ordination to facilitate their functional, and thereby therapeutic, activity. Early indications suggest that pericytes offer an advantage for use in cardiac cell therapy in terms of their robustness to hypoxic insult, making them more equipped to withstand the ischemic microenvironment. However, a much clearer understanding of how pericyte metabolism differs between regions and how metabolism reciprocally interacts with key signaling pathways will undoubtedly provide novel clues as to how we better take full advantage of their therapeutic potential.

## Utilizing Pericytes for Regenerative Therapy

Having established the qualities of pericytes in the ischemic environment, the next challenge is utilizing this potential for a regenerative treatment. This can be approached via two distinct methods: targeting endogenous pericytes by pharmacologic or genetic maneuvers or delivering exogenous pericytes to the damaged tissue. Targeting of endogenous pericytes presents a less invasive option; however, until pharmacological compounds or gene therapy methods specifically targeting pericytes are developed, the approach remains empiric. For example, it is known from the work of Attwell and colleagues that pericyte constriction in the no‐reflow phenomenon can be antagonized with adenosine, calcium antagonists, or endothelin antagonists 81. Unfortunately, these compounds are not pericyte specific and therefore administration can produce undesirable effects in other cell types. While high‐throughput small molecule screening may help to deliver clinically valuable drugs to modulate specific pericyte functions, until this is realized, delivery of exogenous pericytes remains the optimal method of treatment and, therefore, is the focus of the following sections.

### Selecting an Exogenous Pericyte Source

Pericytes have been isolated from several human tissues 22, 59, 82, 83, 84, 85, 86, 87; however, not all of these methods or cell populations are of a clinical grade. For clinical viability, it is important that there is a standardized isolation protocol in place that is minimally invasive for the patient and results in a well‐characterized and highly‐pure cell population. Although a recent phase II clinical trial has demonstrated the safety and feasibility of allogeneic cell therapy in patients with chronic HF 88, autologous cells remain the optimal choice.

Once isolation has been accomplished, pericyte populations are cultured and expanded in vitro until clinically viable numbers have been generated. The process of culturing differs between groups, with many varieties of growth medium and surface coatings used to provide a suitable environment for the pericyte populations 89. Microscopy, flow cytometry, and immunocytochemistry can all be used to achieve a stringent phenotypic characterization of the cell population 12, 13, 15, 22, 89, 90, 91. However, due to the absence of a unique pericyte marker, this alone is not sufficient to distinguish pericytes from similar cells, such as VMSCs. It is therefore crucial to perform a functional characterization of the cells to supplement the antigenic screening as angiogenic assays can identify pericytes from other mesenchymal cells, such as fibroblasts or bone marrow‐derived MSCs, by their enhanced ability to stabilize endothelial networks 92. Table 2 provides a summary of common pericyte populations that have been studied in vitro; however, here we limit the discussion to the pericyte populations that have been explored for potential treatment of CAD, namely pericytes within the heart, vasculature and skeletal muscle, and present the positives and pitfalls of each.

**Table 2:** Summary of commonly explored pericytes with emphasis on their respective identification markers, differentiation potential, and scale up results in vitro

Pericyte subset	Anatomical location	Identification markers^a^	Differentiation potential	In vitro scale up results	References
**Saphenous vein pericytes**	Adventitial vasa vasorum in the great saphenous vein.	Adventitial pericytes in the saphenous vein can be identified by their expression of CD34+/31–.	Differentiation into osteoblasts, adipocytes, myocytes, and neuron‐like cells. No chondrocyte, endothelial or hepatocytic differentiation observed.	Expansion with a doubling time of 45 hours. Potential to reach 50 million cells within 10 weeks. Decelerated proliferation after P10. Pericytes are clonogenic, enhance endothelial networks and release proangiogenic factors in culture. No adverse effects on functionality from cryopreservation or passaging up to P10.	22, 72, 93, 94
**Cardiac pericytes**	Perivascular region around blood vessels in atrial and ventricular myocardium.	Adventitial cardiac pericytes are identified by CD34+/31–/146– expression, while microvascular cardiac pericytes express CD146+/34–/56–/117–. CD117 is a marker of cardiac progenitors, negative gating of this marker allows distinction of microvascular pericytes from cardiac precursors. Cardiac pericytes have also been shown to express cardiac transcription factor GATA‐4.	Induced contractile VSMC phenotype. Partial cardiomyocyte differentiation. Chondrogenic, adipogenic, and osteogenic differentiation potential. Inability for endothelial differentiation or skeletal myogenesis.	Explored in vitro. 20 million cells generated by P5 after 4 to 6‐weeks. Cells remain highly clonogenic with no significant decrease in functionality or phenotypical expression by P5. Functionally they demonstrate angiogenic potential, enhancing endothelial tube networks, recruiting cardiovascular stem cells and producing growth factors and chemokines. No adverse effects on functionality from cryopreservation.	11, 12, 83
**Skeletal muscle pericytes**	Muscle biopsy. Specific location unknown	Microvascular pericytes identified via expression of CD146+/34–/45–/56– and alkaline phosphatase.	Myogenic, adipogenic and neuronal differentiation potential. Minor fraction of skeletal pericytes capable of cardiomyogenic differentiation.	In vitro expansion up to 35 doublings with no alteration of morphology or antigenic profile. Functional characterization in vitro reveals direct and paracrine angiogenic properties. A paracrine antifibrotic effect under hypoxic conditions was also observed.	23, 59, 71, 95
**Cerebral pericytes**	Ventricular zone and temporal neocortex	Pericytes identified using FACS purification of CD13+/CD105+/CD45–/CD31–. This pericyte population also express nestin. Other cerebral pericyte subsets have been isolated based on a co expression of CD73+/CD45– with either high or low CD90 expression.	Capable of typical mesodermal lineage differentiation into osteoblasts, chondrocytes and adipocytes but also harbor neuroectodermal differentiation capacity with differentiation along the glial and neuronal lineages observed.	Highly proliferative in culture with different subsets displaying varied proliferation rates. Cells can be freeze‐stored and thawed without losing proliferation capacity or potency.	86, 96
**Umbilical cord pericytes**	Umbilical cord arteries and vein.	Umbilical cord pericytes are typically identified via CD146 expression, along with CD105+/CD34–/CD45–. A alternative population have also been isolated using an unusual marker array of CD45–/CD34–/SH2+/SH3+/Thy‐1+/CD44+.	Adipogenic, osteogenic, and chondrogenic potential. Osteogenic differentiation capacity lower than similar perivascular cells.	CD45–/CD34–/SH2+/SH3+/Thy‐1+/CD44+ pericytes demonstrate very high expansion potential in culture with a doubling time of 20 hours at passage 2 and 10^10^ cells after 30 days. Concerns over aging of cells and loss of potency in long‐term culture have been reported. Interestingly hypoxic conditions are able to address this aging effect by promoting colony forming efficiency and proliferation, whilst also inhibiting osteogenic differentiation. Cells retain potency following cryopreservation.	21, 85, 97, 98
**Bone marrow pericytes**	Bone marrow cavity of tibia and femurs.	CD146 is used to identify perivascular cells with a pivotal role in vascular niche maintenance. Nestin and α‐SMA expression have also been used for isolation of different bone marrow pericyte populations.	Adipogenic, osteogenic, chondrogenic, and vascular smooth muscle differentiation potential.	Demonstrate the ability to enhance vascular networks in vitro via direct contact and paracrine effects. Display a doubling time of between 3 and 4 days, however, issues regarding limited expansion and senescence in culture have been reported.	29, 99

a Identification markers indicate differing markers expressed by particular subsets of pericytes. Unless otherwise mentioned there is an assumption that subsets also express a typical array of pericyte markers (NG2/PDGFR‐B/αSMA/CD44/CD105/CD90/CD73+, CD31/CD45–).

#### Cardiac Pericytes

Cardiac pericytes (CPs) have been targeted for ischemic heart repair due to their native role in maintaining homeostasis via regulation of microvascular function and angiogenesis 100. Distinct pericyte populations have been isolated from the heart, via selection of conflicting arrays of markers (CD146^+^/CD34^–^/CD45^–^/CD56^–^/CD117^−11^, CD31^–^/CD34^+12^), using fluorescent activated cell sorting (FACS) or magnetic activated cell sorting (MACS). From just 100 mg of human heart tissue, approximately 20 million cells can be generated within 6 weeks 12.

Flow cytometry and immunocytochemistry confirm that CPs display a typical array of pericyte markers 11, 12, although contrary to the isolation protocol, the phenotypic characterization of the CD31^–^/CD34^+^ pericyte population showed them to be CD34^–^ in culture. Functional characterization by Avolio et al. 12 revealed that 10% of CPs were able to form colonies after being seeded into individual wells of a 96 well plate. Furthermore, CPs demonstrated potential for differentiation into a VSMC fate when exposed to inductive medium containing PDGF‐BB. RT‐qPCR demonstrated a respective 18‐fold and 157‐fold increase in expression of α‐SMA and smooth muscle calponin, suggesting a contractile phenotype. Similarly, mature VSMC markers, smooth muscle‐myosin heavy chain and smoothelin, were upregulated by 5.5‐fold and 4.2‐fold, respectively. The CPs were also able to enhance network formation with ECs on a Matrigel substrate, demonstrating their angiogenic ability. In addition, Chen et al. demonstrated the osteogenic, chondrogenic, and adipogenic differentiation potential of CPs 11. This cell population was also able to support EC network formation, stimulate an angiogenic response under hypoxic conditions and demonstrated a limited cardiomyocyte differentiation capacity.

Although phenotypical and functional assays indicate CPs to be a credible candidate for regenerative medicine, the invasive nature of acquiring a biopsy has meant that limited pericyte isolations have been successfully completed. This is because samples can only feasibly be obtained from post‐mortem donors 11, 83, aborted fetuses 11, or discarded tissue from corrective cardiac surgery 12.

#### Saphenous Vein‐Derived Adventitial Pericytes

**Figure 1:**
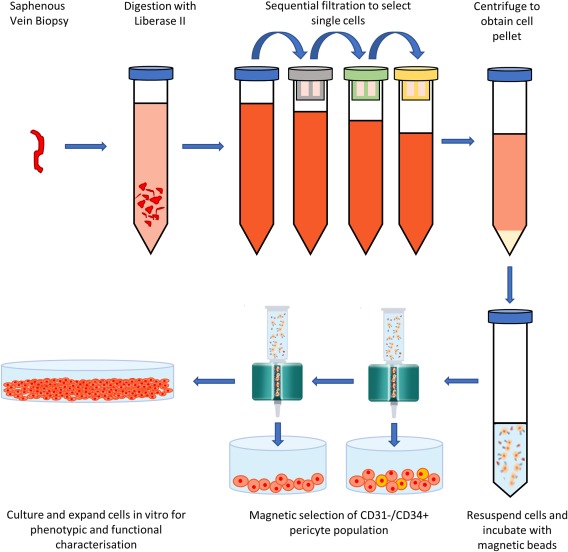
GMP‐compliant isolation protocol. Finely mince saphenous vein biopsy and digest with Liberase II, at 37°C for up to 2 hours. Filter tissue digest sequentially through a 70 μm, 40 μm, and 30 μm mesh to attain a single cell suspension. Centrifuge cell suspension to separate the cell pellet and then resuspended in column buffer. Incubate suspension with CD31 beads for 30 minutes on ice and filter through magnetic column, keeping the CD31– cell population. Repeat incubation and separation with CD34 beads, retaining the CD31–/CD34+ pericyte population. Culture pericytes in EGM‐2 media on culture plastic coated with gelatin and fibronectin.

During coronary bypass surgery, a section of saphenous vein is extracted and transplanted into the patient to relieve the occluded artery. Frequently, there is saphenous vein leftover from surgery that is normally discarded. Importantly, we have shown that this leftover tissue can be used for isolating pericyte‐like cells from the adventitia, referred to as adventitial pericytes (APCs), that possess clonogenic, multipotent, and pro‐angiogenic properties 22. Using either MACS or FACS, CD34+/CD31‐ APCs can be separated from the tissue digest with 70% and 99% purity, respectively. We have worked out that the isolation protocol is compliant with standard GMP procedure (Fig. 1). Importantly, the APC population demonstrated a durability to cryo‐preservation and high expansion capacity, up to passage 10, with a doubling time of 45 hours, allowing generation of 30–50 million cells from a small sample of tissue.

Flow cytometry and immunofluorescent analyses revealed a typical pericyte array of antigenic markers. Intriguingly, the native CD34 expression is downregulated during the culture period, as was also seen in CPs 12. The cells demonstrate a strong clonogenic potential (7%) and differentiation capability into osteoblasts, adipocytes, myocytes and neuronal like cells 22. As expected, these pericytes support capillary network formation when cocultured with ECs on Matrigel. Secretome analysis revealed they support EC proliferation via paracrine mechanisms, particularly through secretion of ANG‐1 22, 72. APCs also secrete pro‐angiogenic microRNA‐132, which is taken up by ECs in coculture and is essential to the ability of APCs to support endothelial network formation 72.

A study by Gubernator et al. evaluated the feasibility of APC expansion for production of a consistent therapeutic cell product 94. APCs demonstrating proangiogenic activities were successful expanded to clinically relevant numbers. The APCs showed a tolerance to extended culture, with antigenic profile and functional properties conserved throughout passaging, and were able to improve blood flow in a model of peripheral ischemia 94. Another important aspect of this study consisted of assessing the association between the APC epigenetic profile, characteristics of the cell donor, and therapeutic outcome. Interestingly, APCs from smoker donors were less effective in improving blood flow recovery compared with cells from nonsmokers. Furthermore, there was a significant inverse correlation between age of the donor and capillary density outcome. It was also shown that the methylation status of a network of genes centered on the VEGFR1 was associated with the effect of APC therapy on microvascular density and blood flow recovery. This suggests that clinical and epigenetic screening may help predict therapeutic response of pericyte therapy. Such an approach would inform the decisions on personalized treatments, that is, the selection of patients who would mostly benefit from the specific cell therapy method.

#### Skeletal Muscle Pericytes

Pericytes of the skeletal muscle present another suitable alternative to the invasive procedure of CP acquisition. These cells can be easily obtained via skeletal muscle biopsies and expanded in culture 13, 59. Using FACS, Chen et al. isolated a CD146+/34–/45–/56– homogenous pericyte population capable of expansion in vitro up to 35 cell doublings 71. When cocultured with ECs on a Matrigel substrate, skeletal muscle pericytes demonstrate the ability to enhance the formation of microvascular networks. In addition, their conditioned medium collected under hypoxic conditions was able to reduce cardiac and muscle fibroblast proliferation, indicating an anti‐fibrotic activity of the secretome 71.

Vono et al. also isolated muscle pericytes using plastic adherence and colony selection based on positive expression of ALP, NG2, and CD146 23. The authors examined the difference between normal muscle pericytes and diabetic muscle pericytes. Although both the diabetic and normal muscle pericytes were able to form networks when cocultured with ECs on Matrigel, the diabetic pericytes formed a less reticulated structure 23. Furthermore, diabetic pericytes possessed a reduced myogenic ability, decreased proliferation rate, and antiangiogenic properties, all resulting from an increased oxidative state. Therefore, risk factors can detrimentally affect the reparative capacity of these cells.

### Pericyte Delivery

As seen with other cell types, the accuracy of delivery and long‐term cell retention remain considerable obstacles for pericyte transplantation. Methods of delivery can fall into two broad categories; direct cell therapy and tissue engineering. For the scope of this review, we limit our discussion to the most promising or widely adopted delivery techniques for pericyte‐based therapy.

#### Direct Cell Therapy

Multiple techniques exist for the direct delivery of cells to the damaged heart, with intramyocardial and intracoronary injection among the most commonly used methods 101, 102. Intramyocardial injection is a surgical procedure whereby cells are directly injected into the myocardial tissue. The assistance of image‐guiding technologies and use of NOGA catheters, allowing left ventricular electromechanical mapping for precise identification of sites of injury, has made this a very accurate procedure, while reducing its invasiveness. However, complications, such as arrhythmias or ventricular perforation at injection sites, still represent a matter of concern 103. It has also been shown, through real‐time visualization, that immediate wash out and venous drainage results in low cell retention, with more cells identified in the lungs than the heart 104. In addition, the increased stiffness associated with the fibrotic heart 105, 106 could negatively affect cell proliferation and differentiation of successfully delivered pericytes, thereby hindering any therapeutic benefit. Despite this, intramyocardial injection remains the most commonly used technique for delivery of pericytes in ischemic animal models 11, 71, 72.

Intracoronary administration of cells is the most clinically practiced of general stem cell delivery techniques 107. Cells are delivered to the damaged myocardial tissue through a catheter inserted into the coronary artery. This technique has the potential to deliver a homogenous distribution of cells to the target tissue 108. However, it is reliant on sufficient perfusion, which is often limited in the ischemic heart, and on efficient homing signals capable of driving cell migration toward the damaged region. As such, several pre‐clinical studies have demonstrated poor cell retention using this technique 109. Furthermore, clinical trials have shown mixed results 110, 111, with at best, only modest levels of clinical benefit being achieved.

An additional consideration for cell therapy is the suggestion that a single dose may not be sufficient for long‐term recovery of chronic CAD 112. In a recent study by Guo et al, repeated dosage of cardiac mesenchymal cells demonstrated a significant increase in cardiac function compared with single dosage 113. A clinical trial (REPEAT—clinicaltrials.gov NCT01693042) is now underway to explore this observation in more detail. If the results of this trial agree with the mentioned study, then the feasibility of repeated pericytes injections will need to be assessed.

#### Tissue Engineering

To overcome some of the issues associated with direct cell delivery, focus has shifted to exploiting the benefits of bioengineering. The growing number of biomaterials suitable for use in the heart, and advancements in additive manufacturing and electrospinning technology, has led to tissue engineering becoming a rapidly developing area.

The typical tissue engineering approach uses a biomaterial scaffold seeded with cells and bioactive factors. By optimizing properties of the scaffold such biocompatibility, biodegradability, porosity, mechanical properties, topography, and biochemical signaling, an extracellular environment can be created that mimics in vivo tissue and positively influences cell proliferation, differentiation, migration, and long‐term engraftment 114, 115, 116. Studies have already demonstrated the feasibility of pericyte‐based tissue engineered vascular grafts for treatment of limb ischemia 117, 118, and animal models using similar cell sources have shown promising results for cardiac repair using scaffold‐based cell delivery 119, 120. That said, there are still some hurdles and challenges to overcome, such as regulations and upscale costs, before this becomes a viable method of cell delivery in the clinic 121, 122.

Hydrogels present an alternative delivery option that capitalizes on the benefits of tissue engineering approaches, in terms of cell retention and engraftment, while remaining less invasive due to their ability to be delivered via injection. Cells are incorporated into a gel which closely resembles natural extracellular matrix, such as collagen, alginate or Matrigel, before being injected into damaged tissue. Studies using animal models have demonstrated the feasibility of hydrogels to deliver stem cells to the infarcted heart 123, 124. Moreover, recent developments toward GMP‐compliant protocols point toward this becoming a possible option for clinical delivery of pericytes in the near future 101, 125.

Overall direct cell delivery methods are still most commonly used for stem cell‐based therapies; however, they are severely limited by poor cell retention and cell survival 126. Studies have shown that after delivery of 100 million cells, usually less than 5% are retained after 24 hours and 99% of these will not survive past the 4–6 week mark 127. While some groups may now be isolating GMP compliant pericyte populations, suitable for therapeutic clinical use, the benefits of these cells will not be fully realized until a more efficient delivery method is established 128. Tissue engineering approaches present a possible solution to this. Even with the relatively new field exploring pericytes as a regenerative cell source, there have been various in vitro and in vivo studies showing the benefits of tissue engineered cell delivery 12, 118, 129, 130.

## Pericytes in Preclinical Models of Ischemic Heart Disease and Translation to Clinic

Although use of pericytes has yet to reach clinical trials, there has been early success reported in studies of animal models.

### Small Animal Models

In 2013, Chen et al. carried out a study on immunodeficient mice with induced MI 71. Mice were injected with either a suspension of skeletal muscle pericytes or a PBS control. Pericyte‐transplantation groups demonstrated significantly better left ventricular contractility, and a 45% reduction in cardiac fibrosis when compared with the control. In addition, pericytes were able to differentiate into cardiac cells and promote angiogenesis. The same group was involved in a study using CPs in the mouse MI model 11. Results showed that a fraction of CPs have cardiomyocytic differentiation capacities; however, recovery parameters were not analyzed.

**Figure 2:**
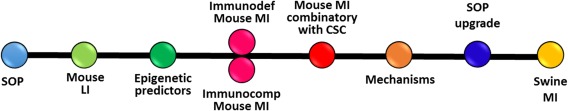
Long‐term strategic plan for clinical translation of adventitial pericytes (APCs). 1. SOP for isolation, expansion, and characterization of highly pure human APCs. 2. Mouse LI model. 3. Identification of epigenetic predictors. 4. Immunodeficient and immunocompetent Mice MI model. 5. Mouse MI model using APCs in combination with cardiac stem cells (CSCs). 6. Identification and study of APCs in vivo angiogenic and therapeutic mechanisms. 7. Upgrade of SOP according to acquired data. 8. Swine MI model. Abbreviations: LI, limb ischemia; MI, myocardial Ischemia; SOP, standard operating protocol.

The success of the in vivo studies presented above are hindered by the reliance on immunodeficient mouse models, with no clear objective to progress into large animal models as part of a transition toward human trials 131, 132. To this purpose, our group have used a strategic plan supporting the translation of saphenous vein APCs from bench‐to‐bedside, through a succession of in vivo studies toward more complex models (Fig. 2).

First, small animal models of limb ischemia have been used to give early identification of APCs in vivo angiogenic capacity and epigenetic predictors of therapeutic efficacy 22. In 2011, we evaluated the therapeutic effects of APCs via intra‐myocardial injection into both immunodeficient and immunocompetent mouse MI models 72. A long‐lasting improvement of cardiac function and increased coronary blood flow was observed with similar beneficial effects reported independently of the immune competence state of the animals. This suggested that APCs are able to modulate the immune response of the recipient thereby opening up the option of using allogeneic or even xenogeneic pericyte populations. In addition, we observed a distinct lack of heart calcification from APCs, in contrast to the 50% calcification seen following BM‐MSC delivery.

Following these results, our group looked at combinatory cell therapy to evaluate whether c‐Kit^+^ cardiac stem cells (CSCs) could aid the effects seen with APCs alone 78. This was one of the few studies that evaluated the advantage of combining different sub‐populations of cells to treat cardiac ischemia, and the only one including pericytes. Echocardiography demonstrated that both cell types individually led to improved contractility, with reduced infarct size and interstitial fibrosis. APCs had a greater angiogenic potential; however, CSCs were superior at promoting cardiomyocyte proliferation and endogenous stem cell recruitment. Although combinatory therapy additively reduced infarct size and promoted arteriogenesis, contractile improvement did not improve beyond single cell therapy. Intriguingly, combination therapy also induced modification of the paracrine properties of the studied cells. Interestingly, secretome analysis following in vitro coculture suggested a complex interaction of the paracrine signaling, which resulted in attenuated secretion of VEGF, ANG‐1, ANG‐2, FGF, and miR‐132, but synergic release of SDF1 78. Understanding these interactions between paracrine signals maybe the key to utilizing a cell free regenerative therapy approach. An additional conclusion to draw from this study is the limitation of intramyocardial delivery. Following combined injection, the two cell populations engrafted distant to one another, likely limiting any communication between the two cell types and, therefore, potential synergic actions.

### Upgrading to Large Animal Models

Comparing results across different models can help achieve a deeper understanding of the findings and enhance the translatability of regenerative medicine. Nevertheless, neither the Food and Drug Administration nor the European Medicine Agency have yet provided clear guidelines on whether cell therapy should be tested in one or more animal species, or how data from different models should be evaluated to justify a clinical trial in humans. Furthermore, an interesting and yet unresolved controversy surrounds the preclinical choice of using human cells or the corresponding animal products, which may better simulate the current allogeneic/autologous approach of clinical cell therapy. To begin responding to this question in relation to pericytes, we recently performed the first study of human and swine APC therapy in a large animal model of acute reperfused MI 93. In vitro cytotoxicity experiments and in vivo engraftment studies indicate rejection of human APCs, due to xenogeneic antigen recognition by swine T cells. This new data contrasts with the apparent tolerance of human APCs by the murine immune system 22, 72. Therefore, we decided to opt for the use of swine cells. An adaptation of the standard operating protocol allowed us to obtain swine cells that showed close similarities with human APCs, as assessed by immunocytochemistry, flow cytometry, and functional assays. Transplantation of swine APCs in a swine model of reperfused MI improved microvascular angiogenesis and interstitial fibrosis, as shown previously in mice with MI induced by permanent occlusion of the main coronary artery, but did not result in improvement in contractility and perfusion. Several factors may account for this discrepancy between studies in mice and swine, with the main factors being the model of infarction (nonreperfused vs. reperfused) and the cell dose, which was scaled up from mice to swine but possibly not enough considering the difference in the heart size between the two species. In summary, the results from pre‐clinical studies support the feasibility and safety of APCs for the treatment of MI. In the large animal model, efficacy appears to be reduced to an improvement of vascularization and reduction of fibrosis, which was still not enough to improve contractile indices. Considering that APC therapy improved contractility in the non‐reperfused model of MI, we speculate that pericyte therapy might be especially amenable to CAD patients not suitable for revascularization.

## Future Considerations

A significant barrier to progress within the field of pericyte based medicine is the lack of standardization. There is still disagreement with what constitutes a pericytes due to the lack of an exclusive and specific marker. This discrepancy has resulted in diverse nomenclature being used to describe the same cell population and variable methodologies of isolations, making it difficult to compare results between research groups 22, 38, 93. We suggest that until a specific marker is identified, a defined nomenclature, marker array and functionality test should be established for pericyte subpopulations. There is also a need for standardization regarding the process of pericyte expansion and purification for clinical use. Although isolation via FACS or MACS often yields a pure cell population, for clinical translation it is essential that there is no contamination and cells behave as predicted. We recommend the use of a phenotypic characterization at an early passage, using immunocytochemistry or flow cytometry, to assess purity. Once a clinically viable passage is reached, the cells should be assessed again phenotypically, but also functionally to evaluate clonogenic, angiogenic, and differentiation potential and ensure culture has not reduced potency or modified expression profiles. It should be noted that since most clinical applications will use frozen stocks of cells, pericyte populations should be assessed from both fresh and frozen sources to check for adverse effects of cryopreservation.

As previously described, some pericyte populations are susceptible to phenotypic changes in culture 12, 22. This instability seems be limited to phenotypic expression, as no genetic instability has been found through several passaging of human APCs 93 and downregulation of CD34 has also been reported in various other cell types as a common artefact of cell culture 133, 134. Nonetheless, before transplanting a cell population into a patient, the effects of mutations must be fully explored to ensure they are not harmful or a factor in tumor formation 135, 136. These in vitro induced phenotypic changes also indicate a pressing need to advance current cell culture practices, which have remained relatively basic over the last decade. Optimization of culture systems to simulate the in vivo stem cell niche may be able to address the phenotypic changes occurring during standard culture practices 37, 137.

In certain environments, pericytes have been identified to play a role in pathogenesis of cardiovascular disease 138, 139, 140. This might counteract the benefit of pericyte‐based therapy. Although authors have attempted to explain the mechanisms behind pericytes role in pathologies, such as fibrosis and calcification 141, there is still a lack of consensus on this important issue 140. Initiation of fibrosis is caused by a cascade of events that result in the activation of collagen producing myofibroblasts. These myofibroblasts deposit pathological ECM resulting in the production of fibrotic tissue. Using genetic fate mapping, pericytes have been identified as a potential myofibroblast progenitor 18, 142. It is hypothesized that signaling cascades, such as TGF‐B and PDGF, cause the detachment of pericytes from vessel walls and subsequent migration and acquisition of a fibroblast‐like phenotype 143, 144. In a rat model of atherosclerosis, it has been demonstrated that pericytes show abundant lipidic vacuoles 144. In addition, the ability of pericytes to differentiate into osteoblasts and chondrocytes, and deposit matrix found in calcified blood vessels suggests that at least some pericyte subpopulations may play a role in vascular calcification 139. Recently, it has also been suggested that pericytes contribute to coronary no‐reflow 81. No‐reflow is a phenomenon whereby microvascular constriction results in ongoing ischemic conditions. In the brain, no‐reflow has been shown to be caused by microvascular pericytes irreversibly contracting the capillaries in response to ischemia 64, 81. A similar mechanism has been proposed for no‐reflow of the heart through the contraction of endogenous CPs. Although there is no direct evidence for transplanted pericytes causing this effect, such a possibility does warrant further investigation. It may be possible that paracrine factors interacting with, or released from, pericytes are responsible for vasoconstriction of coronary capillaries. If this is proven true, the phenomenon may have been responsible for the lack of increased contractility seen in the recent swine MI model reported by us 93. This would also indicate the potential of pericytes as a drug target for the treatment of ischemia as well as chronic hypertension.

It is evident from pericyte‐based animal models of CAD that there is a strong reliance on traditional injection‐based methods for delivery of cells, but these methods are subject to low retention rates and are most probably hindering the therapeutic potential of pericytes as well as other cell types for treatment of CAD. In vivo models would benefit from an evaluation of cell retention and cell endpoints to ensure this is not affecting therapeutic effects. Transition to tissue engineering delivery methods, such as seeding cells in grafts before injecting, may provide the path to realizing pericytes full regenerative potential.

Finally, the use of pericytes for regenerative medicine is still a relatively new area of research and novel therapeutic scenarios are continually emerging. One such scenario is the idea that pericyte secreted exosomes and vesicles could present a possible cell‐free strategy to treat patients with CAD. More studies, particularly in large animals, should be performed to further develop our understanding of pericytes regenerative potential.

## Conclusion

Knowledge of pericyte behavior in both healthy and pathological environments has expanded rapidly in recent years, such that pericytes now present as a promising candidate for treatment of CAD. Pericytes have been isolated from several tissues, with groups beginning to develop and use GMP‐compliant procedures for these purposes 22. Recent studies have also seen research groups' advance from in vitro models to in vivo large animal models. Although tissue repair seems to be mainly related to the ability of pericytes to support reparative angiogenesis, the progress achieved in such a short time is indicative of the exciting potential of pericyte‐based therapies. Nevertheless, before human clinical trials and commercially available treatments can be fully exploited, further considerations should be addressed, with particular emphasis on establishing more large animal studies, developing our understanding of pericytes role in pathologies such as coronary no‐reflow, and advancing our cell culture and patient delivery technology. Initially conceived as a therapeutic approach that would fit in all instances requiring recovery from an injury, cell therapy is recently evolving toward personalized medicine. In this respect, pericytes represent an obvious candidate product for improving vascular growth and perfusion of ischemic tissues, including alleviating the condition of refractory angina patients.

## Author Contributions

W.C.: Literature review, manuscript writing, manuscript revision and figures design. A.F.: Literature review, manuscript writing and manuscript revision. D.M.: Literature review and manuscript writing. P.M.: Manuscript writing, manuscript revision and final approval.

## Disclosure of Potential Conflicts of Interest

The authors indicated no potential conflicts of interest.
